# Laminaria tent use for dilation, extraction, and curettage leading to septic shock: a case report

**DOI:** 10.1186/s13256-025-05238-7

**Published:** 2025-08-20

**Authors:** A. Ferreira, V. Mongrain, L. Mardini

**Affiliations:** 1 Department of Obstetrics and Gynecology, McGill University Health Centre, Montreal, QC Canada; 2 Department of Obstetrics and Gynecology, Hôpital Régional de Saint-Jérôme, Saint-Jérome, Québec Canada; 3 Critical Care Medicine, General Internal Medicine, McGill University, CISSS Des Laurentides, Montréal, Québec Canada

**Keywords:** Laminaria, Extraction, Curettage, Septic shock, Dilation

## Abstract

**Background:**

Laminaria tents are commonly used to aid cervical dilation in gynecological procedures, but their potential risks are not well documented in the literature. This case report describes a severe adverse outcome following their use.

**Case presentation:**

A 27-year-old Caucasian woman G2P1 with a 14 + 1 weeks arrested pregnancy was consented for a dilation, extraction, and curettage. Two laminaria tents were inserted into the endocervix, and the following day the dilation and curettage was performed without immediate complications, although laminaria tents were in an unusual state of disintegration upon removal. Post-procedure, the patient developed fever and pelvic pain, and was found to have positive inflammatory markers, hypoxemic respiratory failure, and disseminated intravascular coagulation. A computed tomography scan revealed multiple septic emboli, and blood cultures grew *Staphylococcus aureus*. The clinical presentation and imaging suggested septic shock and multi-organ failure likely due to retained laminaria tent fragments. A diagnostic hysteroscopy followed by total abdominal hysterectomy and bilateral salpingectomy were performed, which revealed significant endometrial necrosis. The postoperative stay was complicated by diffuse alveolar hemorrhage needing intubation, and infective endocarditis. Despite prolonged antibiotic therapy and surgical intervention, the patient experienced persistent bacteremia but eventually recovered after a 26-day hospital stay.

**Conclusion:**

This case report underscores the risks associated with laminaria tents, including severe infection. Although laminaria tents are typically used for short procedures, their use in this case led to catastrophic outcomes, highlighting the need for careful consideration of prophylactic measures and the potential benefits of antibiotic prophylaxis. This case report describes a patient who suffered from severe complications from laminaria tent insertion.

## Background

Laminaria tents are commonly used to aid cervical dilation in gynecological procedures, including preparation for dilation and curettage. There are limited data available on their potential risks and the severity of possible complications. The use of antibiotic prophylaxis can vary among physicians and is not well documented in the literature [1, 2].

## Case presentation

A healthy 27-year-old Caucasian woman G2P1, with one previous uncomplicated cesarean delivery and otherwise unremarkable past medical history, presented to the hospital with a 14 + 1 weeks arrested pregnancy. After discussion, the patient was consented for a dilation, extraction, and curettage. The day before the procedure, two laminaria tents were inserted in the endocervix without any complications and without any antibiotic prophylaxis. The next day, the laminaria tents were removed and showed a cervix dilated to 14 mm. To be noted, the laminaria tents were in an unusual state of disintegration upon removal. The dilation, extraction, and curettage was performed successfully.

Five days later, the patient presented to the Emergency Department with fever, pelvic pain, positive inflammatory markers, lactic acidosis, refractory hypotension, hypoxemic respiratory failure, and disseminated intravascular coagulation. She was also found to have a right deep vein thrombosis and ischemic hepatitis. Her presentation was in keeping with septic shock and multi-organ failure. A computed tomography (CT) scan of her chest demonstrated multiple septic emboli and blood cultures came back positive for *Staphylococcus aureus*. She was then started on Tazocin and clindamycin, and a pelvic ultrasound was requested. A pelvic ultrasound was requested and showed a right ovarian vein thrombosis extending to the inferior vena cava. The scan findings, clinical picture, and increasing inflammatory markers led the gynecology team to suspect septic shock and multi-organ failure due to remaining tissues or laminaria tent fragments in the uterus, the uterus being the source of *Staphylococcus aureus* infection that propagated to the blood. 

Given the severity of the toxic shock and multiple organ involvement, a multidisciplinary approach led to a decision to perform initial diagnostic hysteroscopy, then total abdominal hysterectomy and bilateral salpingectomy (TAH-BS) as needed. The patient and her partner were consented for the surgery. During hysteroscopy, the uterus was found to be actively bleeding, with necrotic parts. TAH-BS was necessary and showed extended endometrial necrosis. The pathology report described endometrial tissues almost completely disappeared and replaced by granulation tissue and fibrinoleukocyte exudates. There were also micro-abscesses in endometrial glands and superficial myometrium inflammation. 

After the surgery, the patient started to improve clinically; however, on postoperative day 4, the patient was re-intubated owing to diffuse alveolar hemorrhage secondary to septic emboli. Given the persistent bacteremia, a cardiac echo was performed and confirmed the diagnosis of infective endocarditis. The patient remained with bacteremia for a total of 14 days despite early source control and adequate antibiotic coverage. She then recovered and was discharged after 26 days of hospitalization.

## Discussion and conclusion

Laminaria tents should only be used when indicated to dilate and ripen the cervix before completing a dilation and evacuation [2–4]. Some factors will influence the complication rates, such as operator skill and experience and patient characteristics (immunosuppression, previous pelvic infection, anatomy, condition of the cervix, prior surgical interventions, etc.). To reduce the incidence of complications, some studies suggest using adjuvant mifepristone or misoprostol rather than using laminaria dilators alone [4].

As demonstrated, a more advanced gestational age has an increased risk of complications. The prevalence of complication is low but includes bleeding, uterine perforation, cervical injury, pain, or even anaphylaxis. Approximately 5–10% of cases will have failed dilation or displacement or expulsion of laminaria [7].

There is also a potential risk for infection, and ascending colonization from the vagina to the cervix with the use of laminaria. Although infections are rare, the short duration of laminaria placement can help decrease the risk of infection, and the most critical factor in preventing infection has been shown to be an aseptic technique during the procedure [5].

Practices on antibiotic prophylaxis vary among physicians and settings. In the literature, routine use of antibiotics before laminaria insertion is not recommended because the risk of infection is low and not consistently reduced by antibiotics [1, 8]. However, antibiotic prophylaxis should be considered in selected high-risk patients such as those with history of pelvic infections, during second-trimester procedures, or immunocompromised patients [2, 7].

In this case report, the laminaria tents used for a usually short and simple procedure caused severe complications that led to a total abdominal hysterectomy and bilateral salpingectomy, and a prolonged hospital stay of 26 days. Other complications included pulmonary septic embolism, endocarditis, and persisting bacteremia with multi-organ failure. The only risk factors of this patient for complications were the arrested pregnancy at 14 + 1 weeks and the previous cesarean section. Prophylaxis antibiotics were not used considering the low risk of infection. However, in the end, what was thought to be a simple laminaria dilator to help a procedure ended up causing more harm than good.

In conclusion, laminaria tents have a low risk of complications, and antibiotic prophylaxis is usually not indicated except in high-risk patients, where it should be considered. It is also crucial to use an appropriate aseptic technique, and close follow-up after the procedure should be organized with the patient [6, 8].

## Data Availability

Not applicable.
